# Faculty and applicant perceptions of virtual interviews on subspecialty fellowship match in obstetrics and gynecology

**DOI:** 10.1080/10872981.2022.2068993

**Published:** 2022-04-26

**Authors:** Abigail Armstrong, Lindsay Kroener, Joshua G. Cohen, Christina S. Han, Victor W. Nitti, Radhika Rible, Kathleen Brennan

**Affiliations:** a Division of Reproductive Endocrinology and Infertility, Department of Obstetrics and Gynecology, University of California at Los Angeles, Los Angeles, California, USA; b Division of Gynecologic Oncology, Department of Obstetrics and Gynecology, University of California at Los Angeles, Los Angeles, California, USA; c Division of Maternal-Fetal Medicine, Department of Obstetrics and Gynecology, University of California at Los Angeles, Los Angeles, California, USA; d Division of Female Pelvic Medicine and Reconstructive Surgery, Department of Obstetrics and Gynecology, University of California at Los Angeles, Los Angeles, California, USA; e Division of Family Planning, Department of Obstetrics and Gynecology, University of California at Los Angeles, Los Angeles, California, USA

**Keywords:** COVID-19, fellowship, virtual interview, selection process, OBGYN

## Abstract

**Background:**

In response to COVID-19, the AAMC recommended that hospitals conduct interviews in a virtual setting.

**Objective:**

To evaluate whether fellowship video conference interviews (VCIs) are an acceptable alternative to in-person interviews from both the applicant and program perspectives.

**Methods:**

Applicants and faculty from a single academic institution with five OBGYN subspecialty fellowship programs were invited to complete surveys regarding their experience using VCIs during the 2020 interview season. Survey responses used a 5-point Likert scale (strongly disagree to strongly agree). Comparative analyses between faculty and applicants responses to survey questions were performed with two-tailed Student’s t-tests.

**Results:**

45 faculty members and 131 applicants received the survey. Response rate for faculty members and applicants was 95.6% (n = 43) and 46.6% (n = 61), respectively. Faculty and applicants agreed that the VCIs allowed them to accurately represent themselves (83.7% vs. 88.6%, p = 0.48). Most applicants (62.3%, n = 38) reported a fundamental understanding of the fellowship’s culture. The majority of applicants (77.1%, n = 47) and faculty (72.1%, n = 31) agreed that they were able to develop connections during the virtual interview (p = 0.77). Faculty and applicants stated that VCIs assisted them in determining whether the candidate or program, respectively, was a good fit (83.7% vs. 67.2%, p = 0.98).

**Conclusions:**

The VCI fellowship recruitment process allowed OBGYN fellowship applicants and programs to accurately represent themselves compared to in-person interviews. Most applicants and faculty were able to develop relationships over the virtual platform. Although not explicitly assessed, it is possible that the virtual interviews can achieve a suitable match between applicant and program across all OBGYN subspecialty fellowships. The VCI process may be a long-term resolution to minimize both the financial burden and time commitment presented by traditional in-person interviews. Follow-up studies should assess the performance of the virtually selected fellows compared to those selected in previous years using traditional in-person interviews.

## Introduction

While trepidation preceded the 2020 fellowship application cycle due to the COVID-19 pandemic, inefficiencies in the application system predate the global pandemic. Each year, approximately 40% of graduating OBGYN applicants apply to fellowship training programs [1]. For both applicants and programs, the fellowship match process requires significant resource investment. Beyond travel and financial expense, candidates must coordinate their interviews and clinical responsibilities while residency programs are challenged with unpredictable applicant absences. In the setting of multiple subspecialty candidates interviewing during the same academic year, this poses a significant pressure on residency programs to ensure coverage for patient care.

The costs incurred by applicants applying to fellowship is substantial, especially considering the large number of interviews an applicant must attend in order to be successful in the match process. The median salary for a fourth-year applicant across the country in 2019 was $63,982, while matched applicants spend an average of $5,286 per interview cycle [1–3]. Costs are exacerbated by the fact that programs in the same geographic areas may not routinely coordinate their interview dates. In fact, 72% of applicants needed to travel to the same city more than once [4]. Video conference interviews (VCIs) offer an equitable and efficient means for fellowship interviews that may help to overcome some of the hurdles of traditional interviews.

Recognizing the financial and geographic constraints prior to the COVID-19 pandemic, there had been a ‘Call to Action’ for OBGYN programs to improve the subspecialty interview practice by regionally coordinating interview dates or by performing interviews at national meetings in order to create a more cost-effective process [1]. This move toward more coordinated interviews was starting to take hold prior to the 2020 interview season. In response to the public health concerns during the COVID-19 pandemic in 2020, the Association of American Medical Colleges strongly recommended that teaching hospitals conduct all interviews in a virtual setting [5]. Given travel restrictions and the need for physical distancing, all fellowship programs transitioned to VCIs from the traditional face-to-face interviews in 2020.

Despite the potential benefits of VCIs, there is limited data evaluating how virtual interviews specifically affect the satisfaction with the OBGYN fellowship match process. The purpose of this study was to evaluate whether a virtual interview model is an acceptable alternative to traditional in-person interviews from both the applicant and faculty perspectives at a single academic institution. We chose to compare applicants and faculty as these parties represent the two key stakeholders in the fellowship application process. Mutual satisfaction of both parties is crucial for the success or failure of virtual interviews and ultimately the fellowship match.

## Materials and methods

### Study setting, population and evaluation design

All University of California at Los Angeles (UCLA) OBGYN fellowship programs conducted interviews in a virtual setting in 2020. An online survey was created via REDCap to evaluate both the applicant and faculty virtual interview experience. A total of 131 candidates interviewed for five subspecialty programs: the Reproductive Endocrinology and Infertility (n = 21), Maternal-Fetal-Medicine (n = 24), Gynecologic Oncology (n = 29), Female Pelvic Medicine and Reconstructive Surgery (n = 25), and Family Planning (n = 32) programs. A total of 45 faculty members conducted the interviews using the Zoom Video Communications, Inc platform, supported by a team of staff members from the OBGYN Departmental Academic Office.

### Measurement instrument development

Approximately one month after the fellowship match results, applicants and faculty were invited to complete 20 and 13-question surveys, respectively, about their VCI experiences. A 5-point Likert scale was utilized for survey responses, ranging from strongly disagree to strongly agree with an option for neither agree nor disagree. The surveys included questions previously validated from both the Pediatric Surgery and General Urology literature [6,7]. The survey questions aimed to evaluate perceptions surrounding the virtual interview process, ability to form connections and assess efficacy of the virtual process over in-person interviews. The REDCap surveys were distributed via email through the UCLA research department. Participation in the surveys was voluntary and anonymous.

### Data collection and analyses

A limited set of relevant demographic data was collected. We hypothesized that both applicants and faculty would prefer in-person interviews and thus have low satisfaction with the virtual interview process. In order to measure the satisfaction of the virtual interview process, comparative analyses between faculty and applicant responses to survey questions were performed with two-tailed Student’s t-tests. Statistical significance was set at p < 0.05. Institutional review board approval was obtained (UCLA IRB#20-001898). REDCap was provided by the UCLA Clinical and Translational Science Institute grant support (CTSI Grant UL1TR001881).

## Results

Data are summarized in Table 1. 45 faculty members and 131 applicants received the survey. The response rate for faculty members and applicants was 95.6% (n = 43) and 46.6% (n = 61), respectively. Applicants applied to a range of 4–60 programs with an average of 29.2 (±15.2) and were offered an average of 17.8 interviews (±9.62).

**Table 1.** Survey response summary data

	Strongly agree	Agree	Neutral	Disagree	Strongly disagree	P-value
The video conference interviews allowed me to accurately represent who I am						
Applicants	14 (23.0%)	40 (65.6%)	4 (6.6%)	2 (3.3%)	1 (1.6%)	0.48
Faculty	9 (20.9%)	26 (62.8%)	6 (14.0%)	0 (0%)	1 (2.3%)	
I had a good understanding of the program’s culture						
Applicants	7 (11.5%)	31 (50.8%)	14 (23.0%)	9 (14.8%)	0 (0%)	N/A
Faculty	–	–	–	–	–	
I was able to form connections over video conference interviews						
Applicants	12 (19.7%)	35 (57.4%)	5 (8.2%)	8 (13.1%)	1 (1.6%)	0.77
Faculty	3 (7.0%)	28 (65.1%)	6 (14.0%)	6 (14.0%)	0 (0%)	
The video conference interviews helped me decide if the program (if the candidate) was the right ‘fit’ for me (our program)						
Applicants	8 (13.1%)	33 (54.1%)	11 (18.0%)	9 (14.8%)	0 (0%)	0.98
Faculty	8 (18.6%)	28 (65.1%)	5 (11.6%)	2 (4.7%)	0 (0%)	
The video conference interview was worth the time spent						
Applicants	26 (43.3%)	25 (41.7%)	8 (13.3%)	1 (1.7%)	0 (0%)	0.96
Faculty	19 (44.2%)	18 (41.9%)	5 (11.6%)	1 (2.3%)	0 (0%)	
I believe video conference interviews negatively affected the match						
Applicants	2 (3.3%)	5 (8.2%)	17 (27.9%)	21 (34.4%)	16 (26.2%)	0.94
Faculty	0 (0%)	1 (2.4%)	19 (45.2%)	13 (31.0%)	9 (21.4%)	
I prefer video conference interviews to on-site interviews						
Applicants	7 (11.5%)	17 (27.9%)	18 (29.5%)	14 (23.3%)	5 (8.2%)	0.06
Faculty	2 (4.7%)	8 (18.6%)	14 (32.6%)	17 (39.4%)	2 (4.7%)	

^a^P-value: represents the comparison of applicant and faculty responses

Faculty and applicants agreed or strongly agreed that the video conference interviews (VCIs) allowed them to accurately represent themselves (83.7% vs. 88.6%, p = 0.48). 4.9% (n = 3) of applicants did not feel that they could represent themselves accurately during VCIs with the remaining 6.6% (n = 4) feeling neutral. Most applicants (62.3%, n = 38) reported a fundamental understanding of the fellowship’s culture. The majority of applicants (77.1%, n = 47) and faculty (72.1%, n = 31) agreed or strongly agreed that they were able to develop connections during the virtual interview. Faculty and applicants stated that the virtual model assisted them in determining whether the candidate or program, respectively, was a good fit (83.7% vs. 67.2%, p = 0.98). Of the respondents, 85.0% (n = 51) of applicants and 86.1% (n = 37) of faculty strongly agreed or agreed that the VCIs were worth the time spent (p = 0.96). In the applicant group, 82.0% (n = 50) felt comfortable ranking UCLA based on their VCIs. The minority of applicants (14.8%, n = 9) matched at their home institution. A small minority, 11.5% (n = 7) of applicants, believed that VCIs negatively affected their match, whereas 27.9% (n = 17) applicants remained neutral on the VCI process. Only 2.4% (n = 1) of faculty believed that VCIs negatively affected their candidate match, with 45.2% (n = 19) of faculty remaining neutral. There was no significant difference between faculty and applicant preference for VCIs (23.3% vs. 39.4%, p = 0.06).

## Discussion

We believe our study is the first to assess whether the videoconference fellowship recruitment process allowed OBGYN applicants and programs to accurately represent themselves. Our research demonstrates that applicants and faculty alike felt that this mode of virtual interviewing allowed them to make connections. Traditional in-person interviews necessitate a significant amount of resources such as time, financial cost, residency coverage and coordination of travel. The VCI process, therefore, is a practical and sustainable alternative for fellowship interviews. This complements the prior body of literature which advocates for changes in the interview process. However, there is a group of applicants (32.8%) who did not feel VCIs helped them determine whether a program was a good fit for them. It is likely these fellows had never even visited their match programs in-person. It would be interesting to reassess program perceptions amongst this group of fellows as they undergo their training. It is essential to optimize the video conference model so that both candidates and programs are confident that this type of interview will reflect the same match outcomes of a traditional in-person interview.

The COVID-19 pandemic has impacted all aspects of medical training including fellowship interviews. In particular, videoconferencing has become widespread, including transition to web-based national conferences, significant expansion of telehealth visits, video education for trainees and tele-mentoring for surgical techniques [8]. Even prior to the COVID-19 pandemic, one study found that 81% of gastroenterology applicants felt videoconference interviews met or exceeded their expectations with 87% of applicants desiring videoconference interviews as a continued option [9]. Similarly, a study from a surgical oncology fellowship concluded that virtual interviews resulted in a more favorable perception of the interview flow without a difference in overall impression when compared to in person interviews [10]. Additionally, a study performed by an adult reconstruction fellowship observed that 85% of applicants felt they were able to present themselves to their satisfaction [11]. Our results agree with these prior studies and demonstrate that applicants and faculty could accurately represent themselves and form relationships over VCIs. The overwhelming majority of interviewees and faculty agreed that the VCIs were worth the time spent. Most applicants created their rank list based on their VCIs and felt comfortable ranking our institution after their virtual experience. Very few respondents felt the VCI process negatively affected their match. Although not explicitly assessed in this study, it is possible that the virtual interviews can achieve a suitable match between applicant and program across all OBGYN subspecialty fellowships. Overall, this public health-imposed trial of virtual interviews demonstrated that VCIs may alleviate some of the potential difficulties posed by in-person interviews.

There are several limitations to our study. Our study was a cross-sectional observational study and therefore lacks the ability to determine a causal pathway; thus we cannot definitively state that there were no differences in our survey comparisons. Our study was performed at a single institution with a relatively small cohort of applicants and faculty, lacking representativeness and thus generalizability of our findings. We would therefore advocate for future studies to be performed on a national level. Our survey completion rate (95.6% of faculty and 46.6% of applicants) was overall impressive. However, 98 applicants needed to respond to the survey in order to achieve a 95% confidence interval in a population of 131 applicants [12]. As with the design of surveys, these studies inherently have recall and non-response bias. The majority of applicants matched outside their home institutions (85.2%, n = 52). Due to the anonymity of the survey, we were unable to report specific match outcomes between applicants and programs.

Given the practicality of our results, future research across multiple medical institutions and departments is recommended. A follow-up study of faculty and applicant match result satisfaction will help determine if VCI is representative of actual experience in fellowship and if the virtual a priori perceptions truly reflect candidate quality. Prospective studies should establish a uniform, evidenced-based approach for the virtual interview model. Furthermore, future research should evaluate the effect of VCIs on other areas of medical training including the residency and medical school interview process.

The COVID-19 pandemic has rapidly changed the medical training interview process. Our study demonstrates that VCIs are an appropriate and reasonable strategy for fellowship subspecialty interviews. VCIs may be an efficient and equitable long-term alternative to the traditional in-person interviews.

## Acknowedlgements

This study is based on previous work that was presented at the American Society for Reproductive Medicine conference in 2021.
